# Fascial Nomenclature: Update 2024

**DOI:** 10.7759/cureus.53995

**Published:** 2024-02-11

**Authors:** Bruno Bordoni, Allan R Escher, Fabio Castellini, Joanna Vale, Filippo Tobbi, Luigi Pianese, Marco Musorrofiti, Enricomaria Mattia

**Affiliations:** 1 Physical Medicine and Rehabilitation, Foundation Don Carlo Gnocchi, Milan, ITA; 2 Anesthesiology/Pain Medicine, Houston Lee Moffitt Cancer Center and Research Institute, Tampa, USA; 3 Osteopathy, Body Lab Clinica di Osteopatia, Milano, ITA; 4 Osteopathy, Post-Graduate Osteopathic Institute, Lesignano de' Bagni, ITA; 5 Physical Medicine and Rehabilitation, 3C+A Health and Rehabilitation, Roma, ITA; 6 Rehabilitation Medicine, Università eCampus, Milano, ITA; 7 Physical Medicine and Rehabilitation, Centro di Rilievi Nazionale di Diagnosi e Fisioterapia, Caserta, ITA

**Keywords:** chiropractic, osteopathic, osteopathy, connective tissue, fascial, myofascial, fascia

## Abstract

The fascial system is the focus of multiple scientific disciplines, and its nomenclature is debated. What tissue should fall under the definition of fascia? Considering university anatomy books where what is considered connective tissue is described as a fact, and through the science of embryology, which allows us to identify the origin of different body tissues, the article reviews and updates the fascial nomenclature. The text is not a point of arrival but rather a basis from which to start again, with the aim of understanding the function of the fascial continuum in the living. The history of fascial nomenclature in historical and modern contexts is reviewed, including the scientific perspective of the Foundation of Osteopathic Research and Clinical Endorsement (FORCE) organization. The latter has no profit-making purposes and does not hold any copyright.

## Introduction and background

The human body is a set of a variety of structures that create multiple interconnected networks so that everything is aware of the adaptation and responses of different metabolic, mechanical, and electrical environments, creating a status quo of health. When one of these structures "disconnects" from the others due to illness, trauma, and advancing age, the body loses its maximum ability to adapt resulting from stressors, orienting homeostasis towards pathology [1-3].

The connective tissue or fascia is the subject of constant study and investigation. In the surgical field, it is necessary to know the layers that constitute the specific area of the operation to avoid iatrogenic damage; to give an example, from a surgical view, the temple area alone is divided into 13 layers, from the skin to the periosteum [4]. Knowing the different depths of the tissues allows the surgeon to anesthetize an anatomical region correctly or help the patient reduce post-surgery pain. For example, with acetabular fracture the clinician injects an anesthetic drug using fascial areas (fascia iliaca compartment block or quadratus lumborum block), after spinal anesthesia for surgery; in this way, the patient better tolerates post-surgery pain [5].

The connective tissue unites different anatomical structures that are not always in direct contact to share multiple functions. The pterygomandibular raphe connects the buccinator muscle, the deep temporalis tendon, and the superior constrictor of the pharynx muscle [6]. The union of these muscular and tendon structures allows the creation of a functional complex to influence and regulate swallowing and chewing.

Studying the mechanical tension produced by the connective tissue is essential to understanding the cause of a symptom or dysfunction of body movement and allows following an adequate therapeutic procedure. An alteration in the structure and morphology of the plantar fascia can decrease the medial longitudinal arch and become the cause of plantar fasciitis [7].

Knowing the different biomechanical characteristics of the connective system is imperative when the surgeon must choose the anatomical fascial area to create autografts. Reconstructive surgery in the presence of chronic lesions that cause a rupture of the diaphragm and the need for repair can rely on the use of the autologous fascia lata; equally, for the reconstruction of the anterior cruciate ligament in mini surgery, it is possible to use the fascia lata autograft [8,9].

Connective tissue can become a vehicle for infections and cause increased mortality and morbidity. A recent study has shown that patients with kidney disease are more susceptible to developing infections from the oral cavity (in the presence of chronic tonsillitis, tobacco use, alcohol dependence, poor oral hygiene) towards the deeper fascial layers of the neck, compromising the immune status [10]. The same deep layers of the neck can act as barriers to prevent the passage of infectious substances. The alar fascia or intercarotid fascia of the neck, in connection with retropharyngeal and prevertebral fasciae, if functional, acts as a barrier to prevent the spread of infections or abscesses towards the danger space (posterior to the paravertebral space) [11]. The density of the connective tissue that forms the alar fascia is compact and regular, innervated, and vascularized [11].

The fascial system can be a source of pain, not only due to the presence of the passage of nerves to other body structures but also due to the innervation of the connective tissue and the presence of mechanical receptors. Free nerve endings contained in the thoracolumbar fascia can send nociceptive information, mimicking a non-specific lumbar dysfunction; the same post-training muscle pain or delayed onset muscle soreness (DOMS), according to a recent study, could derive in greater percentage from fascial tissue, compared to muscle tissue [12,13].

At the microscopic level, fibroblasts and telocytes are ubiquitous fascial structures that secrete biochemical substances and which may affect local or systemic metabolism. For example, these cells can produce multiple pro-inflammatory substances, alter tissues with which they come into contact (via cellular extensions), or activate silent immune substances, creating a pathological status in the gynecological field and negatively influencing women's fertility [14].

From most current research, fascial tissue is understood as firm, dense, or loose tissue; a structure that connects and covers vessels, nerves, and muscles. In reality, we forget that connective tissue comes in different shapes and consistencies, solid and fluid, like specialized connective tissue [15]. Using a nomenclature that correctly indicates what fascial tissue is fundamental to understanding the various relationships between all body areas; without this assumption, the result of an experimental study will always be partial, just as a narrative review will always lack adequate assumptions. Furthermore, the clinician will see the patient as consisting only of some portions of the connective tissue, with the danger of missing a more complete overview. This article reviews the definition and nomenclature of tissues that fall or could fall within the context of the fascial continuum.

## Review

History of the fascia

In history, the Egyptians (2,500 BC) observed this tissue inside the human body, probably during the preparation of mummification [16]. The term fascia derives from the Greek alphabet (ταινία), whose meaning is ribbon or band; later, it was the Latins who formulated the plural or fasciae and the singular or fascia [16]. In Western medicine, Vesalius (1543, De Humani Corporis Fabrica Libri Septem) was probably the first anatomist to describe the three-dimensionality of fascial continuity without directly mentioning the term fascia [17].

Officially, the term fascia can only be found in 1615, by Crooke, to describe a membranous tissue capable of connecting and covering [16]. In the following century, 1700, anatomical dissection texts increasingly included the terminology of fascia to indicate a membranous tissue, tendon, aponeurosis and always a tissue related to skeletal muscles [16]. In Simmons' studies from 1780, the concept was highlighted that fascial tissue is ubiquitous in the human body, covering the most delicate structures, such as nerves, viscera, and blood vessels [16].

At the beginning of the 1800s, many scholars began to make a nomenclature of the fascia based on the location, morphology, and functions, and always in relation to the skeletal muscles, such as Motherby and Wallis (1801) and Hooper and Grant (1839) [16,18]. Wilson, in 1851, with his anatomy text (The Anatomist's Vade Mecum: a System of Human Anatomy), was the first scholar to consider the dermis as fascia, excluding the epidermis [16]. The same concept of exclusion of the epidermis as fascia can be found in another anatomy book from 1858, namely Gray's anatomy book [18]. In the book by Wilson and Gray, the fascia is described in layers (from the dermis), creating the habit of considering the body's fascial network as a set of layers; we also find this last vision in the twentieth century. Also, in the second half of the 1800s, Foster (1892) and Dunglison and Dunglison (1876) highlighted the concept of fascial layers [18].

In the first half of the 1900s, the term fascia was understood as a sheet or band, a complex tissue covering and connecting other anatomical structures, such as muscles and organs [18]. The International Committee for Anatomical Nomenclature and the Federative Committee of Anatomical Terminology, in 1983 and 1998, respectively, gave further emphasis on the stratification of the fascia (superficial and deep), identifying the fascial tissue as “sheaths, sheets, or other aggregations of dissectible connective tissue" [18]. New concepts were born, such as fascial planes, fascial systems, and fascial spaces [18]. The anatomists and scholars of these two federations frame the connective tissue during anatomical dissections and not in vivo.

**Figure 1:**
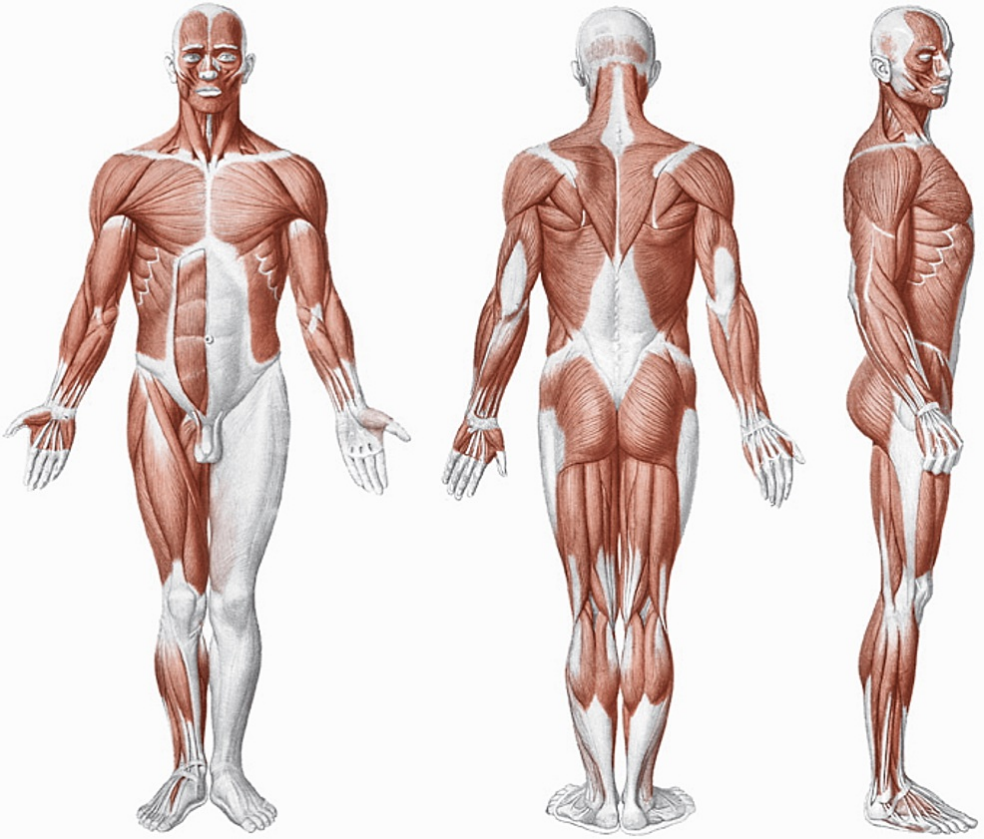
Shape and arrangement of the muscles in the human body The image illustrates the concept of fascial continuity seen only as the connective tissue that covers and interpenetrates the muscles on (a) ventral, (b) dorsal, and (c) lateral surfaces of the human body. This image is reproduced with the permission of Edi-Ermes Publishers, Milano [19].

In 1997, Rosse and Goddum highlighted the concept that connective tissue is connected to all other solid connective tissues in the body, where there is no specific beginning and/or end [18]. Figure 1 shows the shape and arrangement of the muscles in the human body.

Modern history of the fascia

In the 21st Century, fascia has found new vigor in research and clinical interest. In 2007, the first congress on the topic of fascia brought together several scholars and researchers, founding the Fascia Research Congress (FRC); from here, a research group called the Fascia Nomenclature Committee (FNC) was established [16]. FRC/FNC periodically come out with updated nomenclatures on the fascial continuum, the latest of which dates back to 2019: “consists (fascia) of the three-dimensional continuum of soft, collagen-containing, loose and dense fibrous connective tissues that permeate the body. It incorporates elements such as adipose tissue, adventitia and neurovascular sheaths, aponeuroses, deep and superficial fasciae, epineurium, joint capsules, ligaments, membranes, meninges, myofascial expansions, periostea, retinacula, septa, tendons, visceral fasciae, and all the intramuscular and intermuscular connective tissues including endo-/peri-/epimysium. The fascial system surrounds, interweaves between, and interpenetrates all organs, muscles, bones, and nerve fibers, endowing the body with a functional structure and providing an environment that enables all body systems to operate in an integrated manner” [20].

In 2019, the Federative International Program for Anatomical Terminology (FIPAT), which expresses the ideas and knowledge of the International Federation of Associations of Anatomists, described the fascia as follows: “Fasciae/fascia of muscles (deep fascia); investing fascia; fascia of individual muscle (fascia sheath); intermuscular septum; compartment; retinaculum (fasciae of body cavities); parietal fascia; visceral fascia/fasciae; extraperitoneal fascia (extraserosal fascia); extraperitoneal ligament; superficial/deep, middle layer/investing; aponeurosis; membrane; ligament; visceral ligament; tendinous; ring; canal; hiatus; triangle; fat” [21].

In these definitions, the concept emerges that the connective tissue is solid covering and connecting all body areas in a single three-dimensionality. The fascial continuum is no longer a "passive" tissue with respect to the health of the different regions of the body that the fascia unites but actively influences their behavior and health. Furthermore, a connective tissue, defined as specialized adipose tissue, is introduced.

Something is missing

In the scientific literature, there are other tissues that are commonly considered to be specialized and solid fascial tissue. Bone tissue is connective tissue with viscoelastic capacity, innervated and vascularized, with lymphatic pathways and with the same cells (fibroblasts, telocytes) that we can find in the connective tissue that covers and connects muscles and viscera [15,22-28]. Exactly as adipose tissue, specialized connective and equally containing fibroblasts and telocytes (along the vascular pathways), with viscoelastic properties [29-31].

Bone and adipose tissues (white and brown adipose tissue) are not included, compared, and considered in the research together with the "classic" connective tissue; this severely limits the understanding of the fascial continuum in the context of physiological homeostasis (health) and a state of metabolic alteration (disease).

Likewise, if we read anatomy books in university classrooms, for example, chapter 4 (pages 71-84) of Gray's Anatomy (2021), it is highlighted as a fact that blood and lymph fall within the concept of fascial tissue specialized [32]. Blood contains telocytes and fibrocytes (blood-borne fibroblast-like cells) and displays viscoelastic capabilities [33-35].

​We know that there are many studies conducted on the connective tissue that penetrates and envelops skeletal muscles, an anatomical organization that allows muscle contractile fibers to best express mechanical force [36-38]. These studies demonstrate how the presence of "classic" connective tissue facilitates and allows the transmission of force from the muscle in synergy with neighboring muscles. Adipose tissues and blood are equally subject to mechanical forces, as the change in shape due to the passage of mechanical information allows the adaptation and achievement of the function, from the tissue to the cell, up to the genetic response, a mechanism that is at the basis of mechanotransduction [39]. Adipose tissue not only acts as a source of energy, heat, and multiple biochemical substances secreted in paracrine and autocrine modes that interact with metabolism but also acts as a dampener of mechanical tensions [39].

Depending on the tension present, adipose tissue is involved in the production of different tissues; if the mechanical tension felt is congruent with the function of the surrounding tissue, the fat (stem cells) will help the repair and synthesis of the same tissue. In case of an altered perception of the mechanical tension of the surrounding tissues, stem cells derived from adipose tissue could create a non-physiological mechanometabolic environment and lead to dysfunction and disease [40]. A recent study by Hatt and colleagues highlighted that adipose tissue has viscoelastic properties and contributes to the distribution of mechanical loads (in an experimental model); this concept is fundamental to creating biomechanical models that are more congruent with the living being [41]. Currently, we have no in vivo or experimental data on the extent of the presence of fat with respect to the expression of movement, considering all connective tissues simultaneously.

Bone collagen (cortical, trabecular, periosteum) absorbs mechanical energy and is mechanosensitive, varying its mass depending on repeated external stimuli (functional muscle-bone unit) [42,43]. The musculature regulates the function of the bone tissue, and at the same time, the bone exerts multiple mechanical (acts as a lever) and biochemical (transforming growth factor beta, prostaglandin E2, osteocalcin, Wingless-related integration site proteins) influences on the musculature [44,45]. Bone affects the general health of the individual [26]. The bone has skeletal stem cells with the possibility of expressing towards different cell lineages (marrow stromal cells, chondrocytes, osteoblasts, and adipocytes). Currently, we do not have in vivo or experimental data that highlight how much the bone can affect the biomechanics of movement, considering the presence of all connective tissues.

How much do blood (and lymph) and its corpuscular components affect the tension expressed by "classic" connective tissue and other specialized connective tissues? We don't know because blood and other body fluids are not considered when studying, for example, the biomechanics of movement. Yet, not only is it recognized as a connective tissue, but it is a tissue that, like other connective tissues, adapts and responds depending on the mechanical tensions it undergoes [46].

The passage of body fluids between different body regions has a significant impact on the mechanotransductive response of the tissues and cells with which the same fluids come into contact. Direction, velocity, volume, viscosity, and pressure cause specific adaptations. To give an example, depending on the parameters of the tension generated by the passage of blood, the liver can be stimulated to produce certain enzymatic substances. Blood tension triggers the responses of receptors and endothelial cells of the hepatic arterial vessels (stretch-gated ion channels, focal adhesion proteins, cell junction proteins), through the morphological modification of the cellular cytoskeleton [47]. The endothelium thus undergoes a morphological alteration, which activates stretch-sensitive calcium channels; the latter introduces calcium into the hepatocytes, stimulating a further biochemical cascade and the release of liver enzymes [47]. The tissue response is specific to the mode of tension generated by the fluids [48]. The presence of fluids within the bone tissue allows the creation of those fundamental pressures to maintain the mechanotransductive capacity of the bone [49]. Let us remember that blood and lymphatic vessels can also benefit from endothelial stem cells, which help repair the endothelium, even though they are derived from the bone marrow.

The tensional parameters of the CSF (connective fluid with viscoelastic properties) are fundamental for the health of the central nervous system [50]. Cerebrospinal fluid (CSF) can enter the bone marrow via bony channels; from here, it can exit retrogradely, putting the meninges and the medulla (and the nervous system) in mutual contact [51]. The CSF pressure and the information it carries can communicate with specific medullary receptors; the latter will stimulate immune responses that mirror the message received. If the mechanotransductive stimulation is not physiological, the leukocytes produced will invade the nervous tissue, creating an inflammatory environment that, over time, could stimulate the advent of degenerative pathologies [51]. CSF also transports neural stem cells, which can transform into neurons, astrocytes, and oligodendrocytes and for the regeneration of central and peripheral nervous tissue. Body fluids, blood, lymph, glymph, and CSF determine specific pressures with mechanotransductive effects on solid tissues.

Fascial layers from a functional point of view

Every cell in the body is potentially capable of communicating with every other body cell [16]. There are multiple modes of communication between each tissue/cell, such as the formation of nanotubes between cell and cell, in permanent or transient mode; nanotubes can be a few nano millimeters to centimeters long [52]. These tubular and elastic structures comprised of F-actin and myosin Va, bi-directionally transport biochemical, electrical, organelle, and mechanical signals (shortening tension, deformation, stretching) [53,54].

The ramifications of fibroblasts, telocytes, and other cells allow communication with other phenotypically different cells and different tissues in a three-dimensional and continuous context [53]. Furthermore, the constant morphological change of the cell, through a phenomenon known as actin treadmilling (polymerization filaments of actin monomers), allows for constant bi-directional mechanotransductive information [54,55].

The cell can communicate with other cells, via a structure known as primary cilia (1-10 micrometers long, approximately 1 micrometer wide), for the passage of biochemical substances, with the main purpose of transmitting information to obtain a gene response [56].

The cell can input various information into the body fluids (extracellular matrix, blood, lymph, CSF), such as mRNA, microRNA, long non-coding RNA, and DNA, bioactive lipids, receptors, proteins, which substances are inside the extracellular vesicles; the latter are generated inside the cell and released outside and can be divided into exosomes, apoptotic bodies and ectosomes [57].

Another method of cellular communication is quantum tunneling, that is, the crossing of a membrane by electrons, which can interact and stimulate cellular adaptations without prior excitation of the receptors [58]. Every time electrons move and the cell becomes excited, a magnetic field is generated; the change in position or number of electrons generates an electric field, generating an electromagnetic field [59]. The electromagnetic field expands and moves (electromagnetic wave or electromagnetic frequency/oscillation/vibration) and invades the surrounding area, involving other cells in a cascade, putting the whole body in communication [16,59]. This mechanism occurs more easily thanks to the fluid environment of which the human body is made up, and the greater the synchronicity of this phenomenon, the greater the cellular functionality; the speed of information transmission is faster than the electrical neural speed [60].

Regardless of the fascial nomenclature that lists the different layers, the cell communicates with all tissues; knowing the fascial layers to understand the function of the fascial continuum is not the most correct way. The macroscopic or dissectible tissue or fascia is the result of the behavior of the three-dimensional microscopic: the cell. The surgeon emphasizes the dissection respecting the fascial planes but may neglect the general function of the patient's body, that is, the solid and fluid connective tissue that allows the body to function as we see it [17]. And so, it is the same for the researcher or clinician who studies under the microscope or dissects cadavers without having the vision of the body as a whole and the presence of the fluid fascia, respectively.

Anatomy is not only useful to the anatomist, who could be represented as a "photographer" who describes what he sees; it is not only used by the surgeon or doctor in general to make diagnoses. Anatomy is also useful for the clinician who uses manual medicine to understand how to frame the patient. The clinician who employs manual medicine can be represented as an “engineer” who evaluates systemic function. If manual medicine sees anatomy under a functional lens, is it correct that it uses different nomenclature concepts than the anatomist? Maybe yes.

The body cannot and must not be seen as subdivided into planes or axes, as the three-dimensionality of the cells themselves does not respect the measurements dictated by anatomy books. Not only all nervous, vascular, and lymphatic pathways pass through the tissues indiscriminately, without necessarily respecting axes and planes, and considering that these pathways are considered fascial tissue, they too can impact the body's metabolic and mechanical behavior; the extent of this influence is unknown.

The human body is not a text or a data sheet; if it were so simple, everything would be standardized with the same therapeutic effect. But the daily clinic tells us the opposite!

Embryology to understand how to define tissues

Gibson wrote: “...to understand the function it is necessary to study embryology..." [61]. To understand bodily function, it is necessary to study embryology, from which the function developed [17]. Furthermore, the anatomy in books does not always reflect the anatomy of living things, and anatomy itself should be reevaluated starting from ontogenesis [62].

FRC/FNC and FIPAT consider the fascia of the whole body as the solid component deriving from the mesoderm; that's inaccurate. The solid fascia does not arise from the mesoderm alone but also from the ectoderm; connective tissue has a dual phylogeny [16]. Most of the solid fascia of the cranial area derives from the ectoderm (cartilage, fat, connective tissue for muscle tissue, dermis, bones, and more) [63].

To give some examples, the meninges of the skull (dura mater) in the caudal midbrain and forebrain regions arise from the ectoderm, while the remaining dural area derives from the mesoderm [64]. The skull muscles are penetrated and wrapped by connective tissue of ectodermal origin; the ectodermal connective tissue fuses with the mesodermal connective tissue at the level of the muscles of the anterior and posterior craniocervical tract [64]. The parietal bone arises from the mesodermal layer, while the maxillary bone is of ectodermal derivation; the frontal bone is the fusion of the ectoderm and mesoderm [64].

It is impossible to understand the anatomical function without embryology, or if you want to create a nomenclature free from embryological information you risk creating confusion.

Foundation of Osteopathic Research and Clinical Endorsement (FORCE) organization

FORCE is a non-profit organization that has nothing to sell and no copyright on fascial terminology. This organization (since 2013) has the objective of making scientific information updated and useful for manual medicine. It is the only scientific organization of this kind recognized and cited by one of the most important anatomy journals [65].

**Figure 2:**
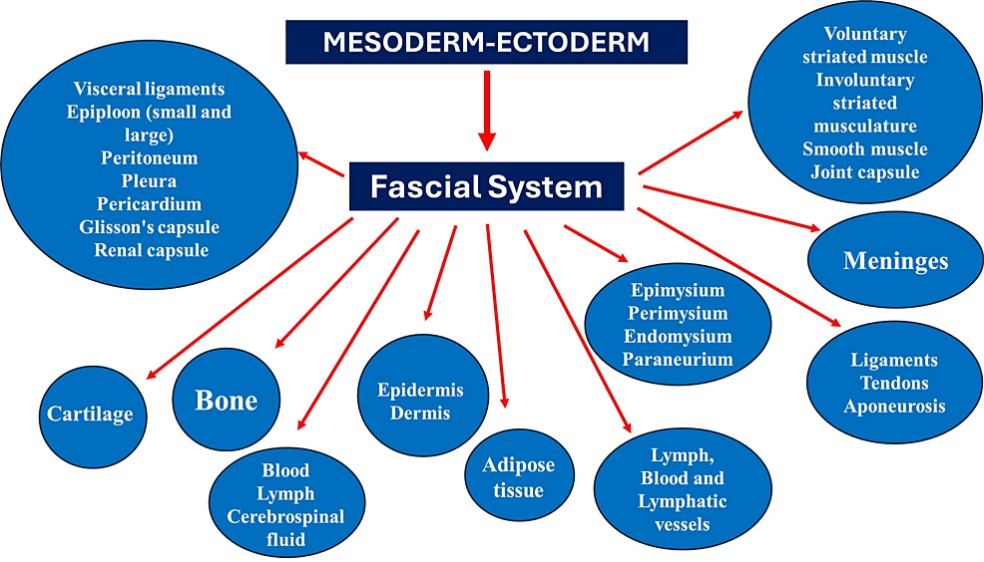
A depiction of fascial tissue The figure summarizes the tissues that the FORCE organization considers as fascial tissue, respecting the dictates of the science of embryology. The image is created by the author Bruno Bordoni.

Our organization considers the solid and fluid fascial continuum deriving from the mesoderm and ectoderm, as already described in previous articles [16,66-68] (Figure 2): “The fascial continuum is the result of the evolution of the perfect synergy among different tissues, liquids, and solids, capable of supporting, dividing, penetrating, feeding, and connecting all the districts of the body: epidermis, dermis, fat, blood, lymph, blood, and lymphatic vessels, cerebrospinal fluid, the tissue covering the nervous filaments (endoneurium, perineurium, epineurium), voluntary striated muscle fibers and the tissue covering and permeating it (epimysium, perimysium, endomysium, *paraneurium*), ligaments, tendons, aponeurosis, cartilage, bones, joint capsule, meninges, involuntary striated musculature and smooth muscle (all viscera derived from the mesoderm), visceral ligaments, epiploon (small and large), peritoneum, *pleura, pericardium, Glisson's capsule, kidney capsule*. The continuum constantly transmits and receives mechano-metabolic information that can influence the shape and function of the entire body.”

Compared to the previous nomenclature, we have added the CSF, which has a double phylogeny, and the coverings of some viscera, such as the pleura and the structure of the pericardium, Glisson's capsule (or Glisson's hepatobiliary sheath), renal capsule (anterior fascia of Gerota, posterior fascia of Zuckerkandl, lateroconal fascia), paraneurium, joint capsule, which structures derive from the mesoderm [67,69-73]. Added connective tissue has been highlighted in italics.

Concluding reflections

A science that will come into play in the future to understand the function of the fascia is quantum biology, as already expressed in previous works [74,75]. Observing the body under the lens of quantum physics will allow us to understand in greater detail cellular behavior, such as connexins and gap junctions, with respect to their role in the development of tumors or the behavior of fibroblasts in the presence of components toxic to the lung [76,77].

We conclude with an article by Lee RP where he highlights the thoughts of the founder of osteopathy, Andrew Taylor Still: “..the fascia, “the framework of life,” is where we live and die” [78]. Furthermore, Dr. Lee underlines the fact that the founder of osteopathic medicine placed connective tissue as an important key in understanding the function of the body and where to find the resources for the patient's self-healing: “Connective tissue is osteopathic tissue” [76]. We should talk about fascial dysfunction and not somatic dysfunction.

## Conclusions

The nomenclature of the fascial system is debated in the literature and currently, we do not yet have an in-depth view of the functions derived from it. Connective tissue can be divided into solid and fluid tissue, interpenetrating each other, making a scenario difficult to decipher exhaustively in vivo. The article reviewed the history of fascial nomenclature and more recent nomenclature from various professional organizations. To conclude, we have updated the nomenclature from a functional point of view of the tissues that could fall within and be considered as fascial tissue through the aegis of embryology, a science that is not relied upon to define the tissues.
